# Repeated Methamphetamine Administration Differentially Alters Fos Expression in Caudate-Putamen Patch and Matrix Compartments and Nucleus Accumbens

**DOI:** 10.1371/journal.pone.0034227

**Published:** 2012-04-13

**Authors:** Jakub P. Jedynak, Courtney M. Cameron, Terry E. Robinson

**Affiliations:** 1 Department of Psychology, University of Michigan, Ann Arbor, Michigan, United States of America; 2 Neuroscience Program, University of Michigan, Ann Arbor, Michigan, United States of America; University of Chicago, United States of America

## Abstract

**Background:**

The repeated administration of psychostimulant drugs produces a persistent and long-lasting increase (“sensitization”) in their psychomotor effects, which is thought to be due to changes in the neural circuitry that mediate these behaviors. One index of neuronal activation used to identify brain regions altered by repeated exposure to drugs involves their ability to induce immediate early genes, such as c-fos. Numerous reports have demonstrated that past drug experience alters the ability of drugs to induce c-fos in the striatum, but very few have examined Fos protein expression in the two major compartments in the striatum—the so-called patch/striosome and matrix.

**Methodology/Principal Findings:**

In the present study, we used immunohistochemistry to investigate the effects of pretreatment with methamphetamine on the ability of a subsequent methamphetamine challenge to induce Fos protein expression in the patch and matrix compartments of the dorsolateral and dorsomedial caudate-putamen and in the ventral striatum (nucleus accumbens). Animals pretreated with methamphetamine developed robust psychomotor sensitization. A methamphetamine challenge increased the number of Fos-positive cells in all areas of the dorsal and ventral striatum. However, methamphetamine challenge induced Fos expression in more cells in the patch than in the matrix compartment in the dorsolateral and dorsomedial caudate-putamen. Furthermore, past experience with methamphetamine increased the number of methamphetamine-induced Fos positive cells in the patch compartment of the dorsal caudate putamen, but not in the matrix or in the core or shell of the nucleus accumbens.

**Conclusions/Significance:**

These data suggest that drug-induced alterations in the patch compartment of the dorsal caudate-putamen may preferentially contribute to some of the enduring changes in brain activity and behavior produced by repeated treatment with methamphetamine.

## Introduction

Repeated intermittent exposure to drugs of abuse produces long lasting changes in behavior, which are believed to be due to alterations in patterns of neural activity within relevant brain circuits [1], [2]. In order to identify areas of the brain activated by drugs of abuse the expression of immediate early genes, such as c-fos or Fos protein, has proved useful [3], [4]. Besides being a marker of neuronal activation, it was recently reported that mice lacking the c-fos gene do not develop locomotor sensitization to cocaine or associated structural plasticity [5]. Thus, alterations in Fos expression might help to identify neural circuits in the brain related to the long-term changes in behavior produced by repeated drug treatment.

One area of the brain that shows changes in Fos expression following repeated treatment with cocaine and amphetamine is the striatum. However, there have been conflicting reports in the literature of tolerance, sensitization, or no effect on striatal Fos expression following repeated drug treatment [6]–[8]. These discrepant findings may be due to a number of factors, such as differences in the environment in which drugs were administered, or the neuroanatomical heterogeneity of the striatum [9], [10]. That is, Fos expression in the striatum can be examined in histochemically distinct compartments: the patch/striosome and the matrix. Neurons in these two areas contain different proteins [11]–[13], they have different afferents and efferents [14]–[16], and have different electrophysiological [17] and structural properties [18], all of which could contribute to differences in neuronal function between compartments. Not surprisingly, repeated drug treatment results in compartmental differences in the pattern of Fos expression. Specifically, repeated administration of cocaine or amphetamines is reported to alter the relative degree of Fos expression in the patch vs. matrix [19], [20].

The purpose of the current study was to examine the effects of repeated methamphetamine administration on Fos expression in specific striatal compartments. However, unlike previous studies, methamphetamine was administered in a novel environment, which is known to produce more robust sensitization compared to injections in the home cage and to facilitate striatal Fos expression [21]–[23]. Furthermore, immunohistochemical techniques were used to investigate drug-induced Fos expression in the patch vs. matrix compartments of the dorsal striatum, and in the ventral striatum or nucleus accumbens. In addition, an automated behavioral analysis system was used to record and analyze different types of drug-induced behaviors to assess the degree of psychomotor sensitization.

## Results

### Effects of repeated methamphetamine administration on psychomotor behavior

**Figure 1:**
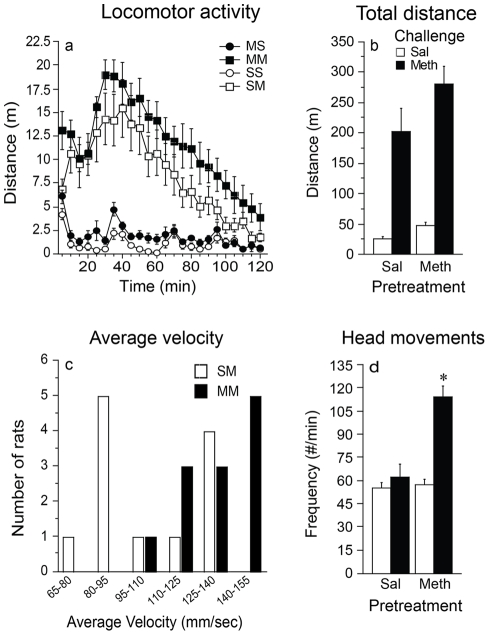
Psychomotor activity as function of treatment and challenge. (a) Locomotor activity in 5 min blocks over 120 min. First letter denotes pretreatment and second letter denotes challenge; S = Saline; M = Methamphetamine (values = Mean±SEM). (b) Total locomotor distance over 120 min (values = Mean±SEM). (c) Number of rats in specific average velocity ranges separated by pretreatment group. (d) Frequency of head movements over 30 min (values = Mean ±SEM). Asterisk denotes significant interaction between pretreatment and challenge.

Figure 1 shows the effect of challenge injection of methamphetamine or saline on locomotor distance travelled (A and B), locomotor velocity (C), and the frequency of head movements (D) as a function of pretreatment condition. Analysis of the time course for distance traveled in Figure 1A, resulted in no significant interaction between pretreatment, challenge, and time [F(23,1012) = 0548; p = 9590], therefore, the data were collapsed over the entire session to simplify analysis. Methamphetamine challenge [F(1,44) = 71.529; P<0.0001)] and pretreatment [F(1,44)  =  4.250; P = 0.0452)] resulted in higher levels of locomotor distance as compared to saline (Figure 1B). However, distance traveled in response to a saline or meth challenge did not vary significantly as a function of past treatment [F(1,44) = 1.458; P = 0.2336)], indicating that this measure did not provide evidence of behavioral sensitization. Exposure to amphetamine can produce behavior dominated by stereotyped actions; therefore, locomotor distance is sometimes not the most sensitive measure of sensitization [24]. For this reason, we also examined other aspects of locomotor behavior, including velocity of locomotion [24]. Figure 1C shows a significant rightward shift in the average velocity of locomotor bouts in methamphetamine challenged rats that were pretreated with methamphetamine versus saline [Fisher's Exact Test = 12.027, *df* = 5, p = 0.012). Another indicator of the intensity of stereotyped behaviors that occurs when animals are not engaged in locomotion is the frequency of lateral head movements. Repetitive head movements were analyzed immediately following methamphetamine and saline administration for 30 min, a time period where stereotyped behaviors are more apparent than locomotor activity. The frequency of head movements in response to methamphetamine or saline challenge varied as a function of pretreatment [F(1,44) = 16.976; P = 0.0002]. Specifically, methamphetamine challenge enhanced frequency of head movements in methamphetamine pretreated animals compared to animals pretreated with saline (Figure 1D; t(22) = 4.751, p<0.0001). Thus, treatment with methamphetamine clearly produced psychomotor sensitization, which was evident both in the velocity of locomotion and the ability of a drug challenge to produce repetitive head movements.

### Effects of repeated methamphetamine administration on Fos expression in the dorsolateral caudate-putamen patch and matrix compartments

**Figure 2:**
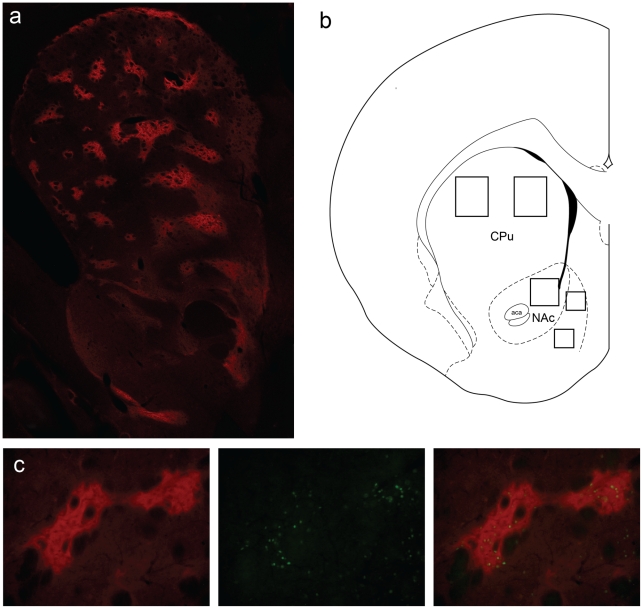
Images of MOR and Fos immunostained tissue. (a) Image of MOR immunoreactivity in the striatum. (b) Placement of templates used for analysis of Fos expression in the dorsal caudate-putamen and nucleus accumbens core and shell. (c) Left, closeup image of MOR immunostained tissue displaying the patch and matrix compartments. Middle, closeup image of Fos expression. Right, overlay of the left and middle images demonstrating Fos expression in patch compartments.

**Figure 3:**
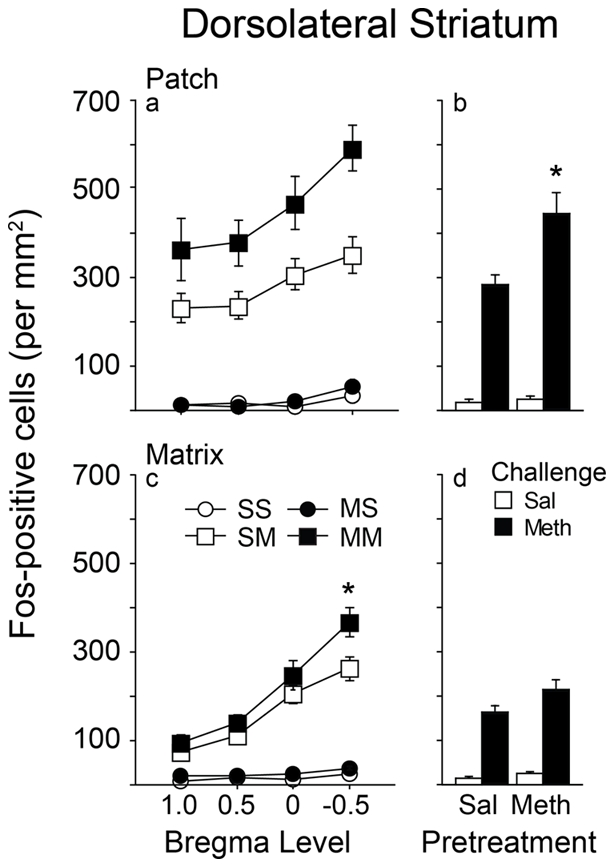
Fos expression in the patch and matrix compartments of the dorsolateral caudate-putamen as a function of pretreatment, challenge, and bregma level. Left panels (a and c), Fos expression across rostral-caudal levels. Right panels (b and d), Fos expression collapsed across levels. Asterisk denotes significant interaction between pretreatment and challenge (values = Mean±SEM).

Examples of mu opiate receptor (MOR) and Fos immunostained tissue along with template placement for Fos analysis in dorsal striatum are shown in Figure 2. A methamphetamine challenge increased Fos expression in both saline and methamphetamine pretreated groups relative to animals challenged with saline, across all rostral-caudal levels in the patch compartment of the dorsolateral caudate-putamen (Figure 3A). Levels of Fos expression were higher in caudal than more rostral levels. There was no significant interaction between pretreatment, challenge, and rostral-caudal level [F(3,123) = 0.662; = 0.5769], so the data were collapsed across all levels to simplify analysis (Figure 3B). The methamphetamine challenge increased Fos expression significantly more in methamphetamine versus saline pretreated animals as indicated by a significant treatment by challenge interaction [F(1,41) = 8.839; p<0.0049].

In the matrix compartment of the dorsolateral striatum, a methamphetamine challenge increased Fos expression in both saline and methamphetamine pretreated groups, relative to a saline challenge, across all rostral-caudal levels (Figure 3C). Similar to the patch compartment, Fos expression was higher in caudal then more rostral levels. A significant pretreatment, challenge, and rostral-caudal level interaction was found [F(3,126) = 2.839; p = 0.0407], due to a significant pretreatment by challenge interaction at the most caudal level [F(1,42) = 4.510; p = 0.0395]. If the data were collapsed across rostral-caudal level there was no significant difference in Fos expression between saline and methamphetamine pretreated groups following methamphetamine challenge [F(1,42  = 2.029; p = 0.1617; Figure 3D], although there was a significant effect of past experience with methamphetamine at the most caudal level examined (Figure 3C). Thus, with the exception of bregma level −0.5, Fos expression in response to methamphetamine challenge was enhanced in the dorsolateral caudate-putamen patch but not matrix compartment of methamphetamine versus saline pretreated animals. Finally, methamphetamine challenge increased Fos expression to a greater extent in the patch compared to the matrix, as indicated by a significant interaction between pretreatment, challenge, and compartment [F(1,368) = 6.339; p<0.012].

### Effects of repeated methamphetamine administration on Fos expression in the dorsomedial caudate-putamen patch and matrix compartments

**Figure 4:**
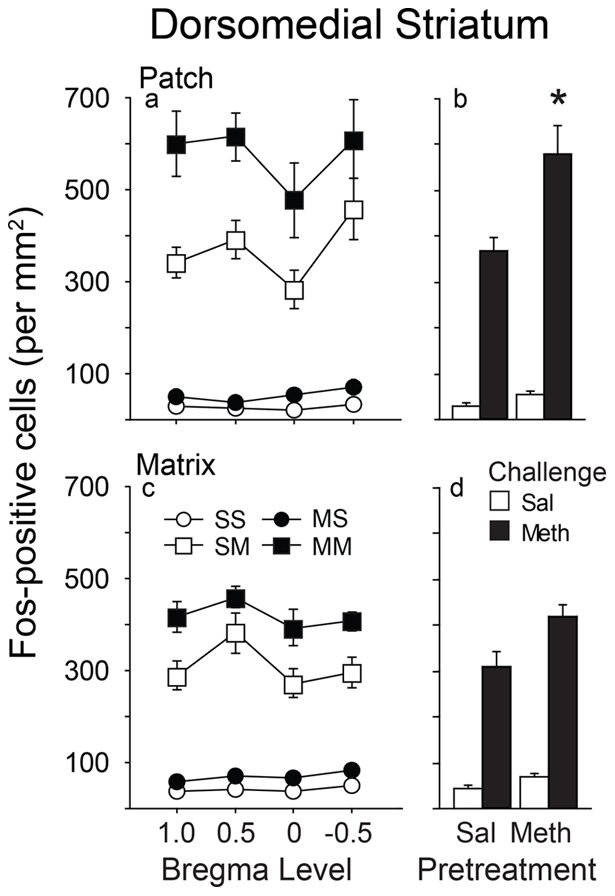
Fos expression in the patch and matrix compartments of the dorsomedial caudate-putamen as a function of pretreatment, challenge, and bregma level. Left panels (a and c), Fos expression across rostral-caudal levels. Right panels (b and d), Fos expression collapsed across levels. Asterisk denotes significant interaction between pretreatment and challenge (values = Mean±SEM).

In the patch compartment of the dorsomedial caudate-putamen, a methamphetamine challenge increased Fos expression in both saline and methamphetamine pretreated animals, relative to a saline injection, across all rostral-caudal levels (Figure 4A). There was no significant interaction between pretreatment, challenge, and rostral-caudal level [F(3,117) = 0.550; p = 0.6491], so the data were collapsed across all levels (Figure 4B). In animals that had been pretreated with methamphetamine, a challenge injection of methamphetamine produced a greater increase in the number of Fos-positive cells than it did in saline pretreated animals, as indicated by a significant treatment by challenge interaction [ F(1,41) = 7.196; p = 0.0107; Figure 4B].

In the matrix compartment of the dorsomedial caudate-putamen, a methamphetamine challenge increased Fos expression in both saline and methamphetamine pretreated groups across all rostral-caudal levels. There was no significant interaction between pretreatment, challenge, and rostral-caudal level [F(3,117) = 0.943; p = 0.4219 ], so the data were collapsed across all levels (Figure 4D). The effect of past experience with methamphetamine on the ability of a methamphetamine challenge to increase Fos expression in this region was not quite statistically significant [F(1,42) = 3.919; p = 0.0543; Figure 4D]. Furthermore, as in the dorsolateral caudate-putamen, methamphetamine challenge induced a greater number Fos positive cells in the patch compared to the matrix (significant interaction between pretreatment x challenge x compartment F(1,363) = 3.934; p<0.048]).

### Effects of repeated on methamphetamine administration on Fos expression in the nucleus accumbens

**Figure 5:**
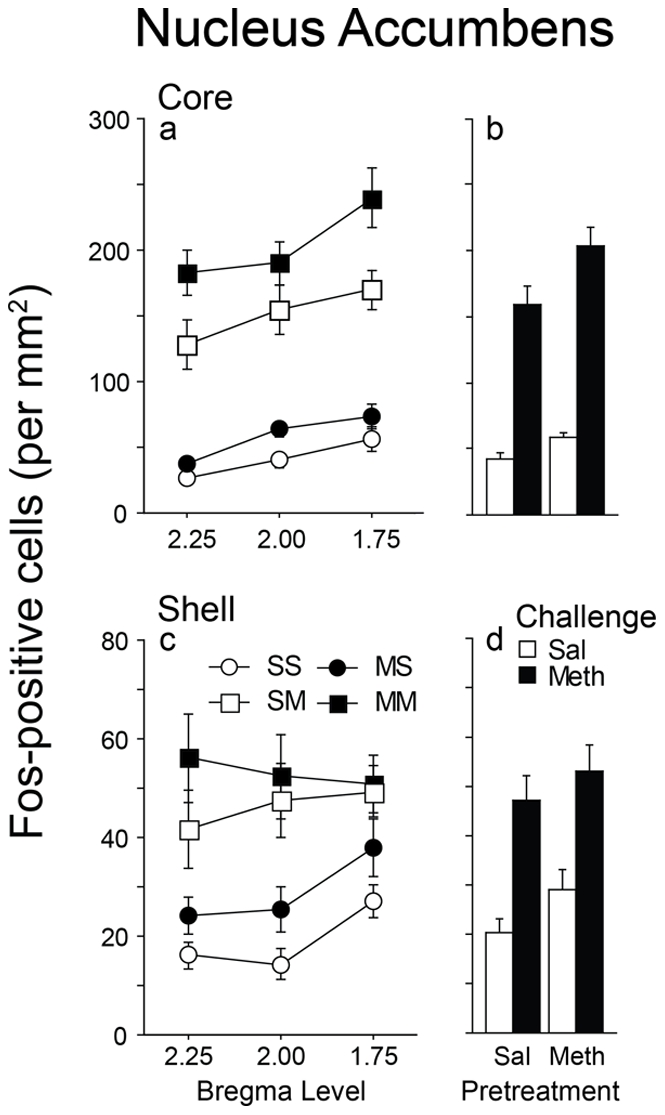
Fos expression in the nucleus accumbens core and shell as a function of treatment, challenge, and bregma level. Left panels (a and c), Fos expression across rostral-caudal levels. Right panels (b and d), Fos expression collapsed across levels. (values = Mean±SEM).

A three-way interaction between pretreatment, challenge, and rostral-caudal level was not significant in the core [f(2,88) = 1.344, p = 0.2661] or shell [f(2,88) = 0.398, p = 0.6727], so data were collapsed across all levels to simplify analysis. In both the core and shell (Figure 5), a methamphetamine challenge increased Fos expression in both saline and methamphetamine pretreated groups, relative to saline challenge, however, there was no effect of pretreatment condition [core: F(1,44) = 1.792; p = 0.1875; shell: F(1,44) = 0.126; p = 0.7239].

## Discussion

We found that repeated methamphetamine administration produced an enhanced psychomotor response upon subsequent exposure to methamphetamine, which is consistent with many previous findings [25]–[27]. Pretreatment with methamphetamine also enhanced methamphetamine-induced Fos expression preferentially in the patch compartment of the dorsal caudate-putamen, relative to both the matrix compartment of the dorsal striatum or the nucleus accumbens core and shell regions. The compartmental specificity of this form of “neural sensitization” may be related to inherent biochemical and/or neuroanatomical differences between the patch and matrix.

The striatum is composed of two histochemically distinct compartments organized in a mosaic fashion. Each compartment can be visualized by examining biochemical markers specific to each area. For example, the patch is rich with MOR receptors [13], [28] whereas the matrix contains high levels of calbindin [11] and acetylcholinesterase [12]. In addition to differences in protein expression, each compartment is differentially innervated by afferents arising from midbrain dopaminergic and cortical glutamatergic neurons. For instance, the patch is predominately innervated by projections from the limbic cortices such as the prelimibic and infralimbic areas [29] whereas the somatosensory cortex innervates the matrix compartment [30]. These differences in biochemical composition and cortical input support the notion that neurons in each compartment possess unique physiological properties. Unfortunately, due to the small volume that the patch compromises of the total striatum, few studies have compared the electrophysiological properties of cells in the patch versus the matrix. Nevertheless, it is reported that cells in the patch have higher resting membrane potential and input resistance [17]. Differences in MOR inhibition of inhibitory post synaptic currents and the ability of NMDA receptors to modulate dopamine release have also been observed between compartments [17], [31]. These neurobiological differences between the patch and matrix could contribute to the compartmental differences in Fos expression observed in this study.

Following repeated exposure to methamphetamine, the ability of a subsequent injection of methamphetamine to increase Fos expression was enhanced in the patch compared to matrix. Preferential activation of the patch vs. matrix in sensitized animals has been reported previously [19], [20]. However, previous studies reported only a *relative* enhancement in patch activation, which was primarily due to a *decrease* in matrix activity, rather than an increase in patch activity [19], [20]. Thus, while others found no change in patch activity following repeated amphetamine administration, we report enhanced patch activation with little to no change in matrix activity. These discrepant findings could be due to a number of differences in experimental design. First, the drug used here was methamphetamine rather than d-amphetamine. Methamphetamine was recently demonstrated to be more effective at releasing DA and increasing internal calcium concentrations relative to d-amphetamine [32], which may be relevant to the drug-induced changes in Fos expression reported here. Second, female rats were used in this study and drug-induced Fos-expression in the striatum has been shown to be sexually dimorphic [33], [34]. Third, in the present study the daily injections were given in a relatively novel and unique test environment, whereas Canales & Graybiel [19] and Vanderschuren et al [20] administered the drug in the animals' home cage. This last point may be especially important because previous experiments from our laboratory have demonstrated that the context in which drugs are administered can have a large effect on the ability of drugs to produce sensitization and induce immediate early gene expression. Badiani and colleagues have reported that psychomotor stimulant drug administration in a novel environment enhances c-fos expression in the caudate putamen and cortex significantly more than when it is given in the home cage [22], [23]. Moreover, the extent of cortical activation differs in animals repeatedly treated with amphetamine in a novel environment compared to those treated in their home cage [35]. Specifically, prefrontal cortical areas such as the prelimbic and infralimbic, which have been shown to predominantly project to the patch compartment, show greater increases in c-fos induction than the somatosensory cortex, which projects to the matrix [35]. These cortical projections release glutamate onto medium spiny neurons, a neurotransmitter that has been shown to be involved in the induction of c-fos in the striatum [36], [37]. Different neuronal populations are also activated when psychostimulant drugs are administered in home vs. novel environments. Specifically, amphetamine administration in the home cage induces c-fos gene expression primarily in the direct pathway or substance p/dynorphin expressing cells, while injections in a novel environment activate direct projections and the indirect pathway or enkephalin containing cells as well [38], [39]. Neuroanatomical studies have shown that the patch contains a higher percentage of direct compared to indirect projections [18], [40] and enkephalin expression is more abundant in the matrix [41]. Therefore, it's possible that injections of methamphetamine in the novel environment recruited indirect pathway neurons in the matrix to overcome the decreases in matrix activation observed by others following repeated amphetamine administration in the home cage [19], [20]. We speculate that the administration of methamphetamine in a relatively novel test environment may enhance neuronal activation in both the patch and matrix compartments relative to when injections are given in the home cage.

Past experience with methamphetamine had no significant effect on Fos expression in the nucleus accumbens. This result was unexpected since recent evidence suggests that Fos expression is enhanced in the nucleus accumbens following chronic cocaine or amphetamine administration in a novel environment [6], [42]. However, others have reported no changes in Fos expression following repeated amphetamine administration although drug was administered in the home cage [20]. As mentioned above, differences in the experimental design could account for these discrepant findings. Moreover, the nucleus accumbens is a heterogeneous structure that can be segregated into territories based on the distribution of various proteins and peptides or differences in afferent and efferent connections [43], [44]). Therefore, it is quite possible that areas within the nucleus accumbens core and shell regions that underwent neural sensitization were missed. A more thorough analysis of the nucleus accumbens with emphasis on inherent biochemical and neuroanatomical differences is warranted.

Repeated exposure to methamphetamine produced psychomotor sensitization and enhanced Fos expression preferentially in the patch compartment of the dorsal caudate-putamen. The localization of Fos sensitization to the patch but not the matrix could have contributed to the behavioral changes produced by repeated methamphetamine treatment. Unfortunately, little is known about the different functional contributions of the patch and matrix compartments to behavior. Some evidence suggests a role for the patch in reward mechanisms since animals will more reliably electrically self-stimulate when the electrode is located in the patch relative to the matrix [45]. Others have demonstrated that enhanced patch activation following repeated cocaine or amphetamine administration correlates with stereotyped motor movements [19]. Another possible role for the patch stems from neuroanatomical studies which demonstrate that limbic structures such as the amygdala, which mediates incentive motivational processes, primarily innervate the patch compared to the matrix [14], [46], [47]. Of course, most studies examining motivation have focused on the nucleus accumbens and related reward circuitry; however, recent evidence suggests that the caudate-putamen might contribute to incentive motivational functions. Specifically, lesions of the dorsal caudate-putamen impair instrumental performance during conditioned stimuli presentation in a Pavlovian-to-instrumental-transfer task [48]. Also, dopamine restoration in the caudate-putamen of tyrosine hydroxylase deficient mice rescues motivated behaviors such as feeding [49], [50]. Clearly, the heterogeneous makeup of the striatum requires more studies to elucidate the role of specific regions and compartments in different behaviors and psychological components of reward [51].

In closing, there is a wealth of information recently available on circuits and compartments within reward-related pathways such as the caudate-putamen or nucleus accumbens that undergo changes at the cellular, molecular and structural levels following repeated drug administration. We report here that repeated treatment with methamphetamine, which produced robust psychomotor sensitization, enhances neuronal activity in the patch but not matrix compartment of the dorsal caudate-putamen. The hypersensitivity of patch medium spiny neurons to subsequent drug exposure might have important implications for the long-lasting behavioral changes observed in animals repeatedly treated with methamphetamine. The challenge now is to understand how drug-induced neuroadaptations in specific neural circuits affect the pathway's overall function and ultimately the behavior of the animal.

## Materials and Methods

### Ethics Statement

All procedures were approved by the University of Michigan Committee on the Use and Care of Animals under protocol number A3114-01.

### Subjects

Forty-eight female Sprague-Dawley rats (Harlan Laboratories; Indianapolis, IN) weighing 220–280 g were housed four per cage in temperature and humidity controlled rooms. They were maintained on a 12-h light:dark cycle with access to food and water *ad libitum.* Animals were acclimatized to these conditions for 7 days prior to testing. Female rats were used because structural and behavioral changes following repeated amphetamine administration have been observed to persist for months following drug cessation [52], [53].

### Groups and test procedures

On treatment days animals were transported from their home cages to activity chambers (33.02× 68.58×60.96 cm) with woven wire grid floors, PVC sides, and cameras (SPE-57, CCTV Specialty Bullet Cameras, Lake Worth, Florida, USA). At the beginning of each session, animals were allowed to habituate to the chamber for 30 minutes before receiving an intraperitoneal (IP) injection of either d-methamphetamine HCl (2 mg/kg, weight of the salt; n = 24) or saline (n = 24). After the 5^th^ pretreatment day, animals remained in their home cages for 7 days during which no drug treatments were given. Following this drug-free period, saline and methamphetamine pretreated rats received a challenge injection of either saline or 1 mg/kg methamphetamine, as during the pretreatment phase, resulting in 4 groups: 1) Rats pretreated with methamphetamine and challenged with saline (MS; n = 12); 2) rats pretreated with methamphetamine and challenged with methamphetamine (MM; N = 12); 3) rats pretreated with saline and challenged with saline (SS; N = 12) and 4) rats pretreated with saline and challenged with methamphetamine (SM; N = 12). Following the challenge injection, behavior was recorded for 120 minutes via a Pelco DX9100 (Clovis, CA) digital video recorder.

### Behavior

Videos captured by the Pelco digital video recorder system were analyzed using TopScan Software (CleverSys, Inc. Reston, Virginia, USA). Behavioral parameters defined specifically for quantification of drug-induced psychomotor activity were used to analyze locomotion and head movements [24]. Locomotion was defined as a forward movement in which the animal traveled a minimum distance of 110 mm. From the locomotor data, distance (mm) traveled and velocity (mm/s) for each locomotor bout was calculated. For analysis, the total distance and average velocity for each movement over 2 h was calculated for each animal. Lateral head movements were defined as deviations of at least 10 degrees from the center of the body when the animal was stationary for at least 2 s. The frequency of head movements was calculated by dividing the total number of head movements by the time spent in place. For analysis, the average frequency of head movements for 30 min following saline or meth administration was calculated for each animal.

### Immunohistochemistry

Two hours after the final challenge injection, rats were deeply anesthetized with sodium pentobarbital (Fatal Plus; Vortech Pharmaceuticals, Dearborn, MI) and transcardially perfused with 150 mL of phosphate buffered saline (PBS; pH = 7.4) followed by 300 mL of 4% paraformaldehyde (PFA; pH = 7.4) at 30 mL/min. Brains were postfixed in 4% PFA at 4°C for 24 h and then transferred to a 30% sucrose/PBS solution for an additional 24 h at 4°C. Following sucrose infiltration, 40 µm coronal sections were prepared on a microtome (Leica Microsystems; Wetzlar, Germany) and stored in wells containing PBS at 4°C. Free-floating sections were washed in PBS for 5 min and placed in blocking buffer (5% normal donkey serum and 0.4% Triton X-100 in PBS) for 1 h at room temperature. Sections were *simultaneously* incubated in primary antibodies goat anti-Fos (sc-52-G; Santa Cruz Biotechnology; Santa Cruz, CA) diluted 1∶500 in blocking buffer and rabbit anti-mu opioid receptor (ab10275; Abcam; Cambridge, UK) diluted 1∶2000 in blocking buffer for 24 h at 4°C. The next day, sections were washed six times for 10 min in PBS and placed in blocking buffer with Image-IT FX signal enhancer (Invitrogen; Carlsbad, CA) for 1 h at room temperature. Sections were simultaneously incubated in secondary antibodies donkey anti-goat (Alexa Fluor 488; Invitrogen) and donkey anti-rabbit (Alexa Fluor 594; Invitrogen) diluted 1∶250 in blocking buffer containing signal enhancer for 2 h. Sections were washed six times for 10 min in PBS, mounted on Fisherbrand Superfrost/Plus slides (Thermo Fisher Scientific Inc., Waltham, MA), and coverslipped with Prolong Gold Antifade Reagent (Invitrogen). See Figure 2 for images of MOR and Fos immunostained tissue. We like to acknowledge that previous experiments examining psychostimulant drug-induced Fos protein expression in the striatum [6], [19] used shorter post-fix lengths (2 h vs 24 h) and different detection methods (DAB precipitate versus immunofluorescence detection) which could have affected the results presented here.

### Quantification of Immunohistochemistry

For each animal, Fos immunoreactivity was quantified in the dorsolateral and dorsomedial caudate-putamen (approximately +1.0, +0.5, 0.0 and −0.5 mm relative to Bregma) and the core and shell subregions of the nucleus accumbens (approximately +2.25, +2.0, and +1.75 relative to Bregma). See Figure 2 for placements of templates in the caudate-putamen and nucleus accumbens. Images were captured at 10×magnification (NA 0.30) using a Leica DMRX fluorescence microscope (Leica Microsystems, Wetzlar, Germany) attached to a Sony DXC-970 MD color video camera (Sony Corporation of America, New York, NY). Images of Fos- and MOR-immunoreactivity were captured and analyzed using MCID software (InterFocus Imaging Ltd., Linton, England). During image acquisition, exposure time was held constant and the signal gain was decreased until the background pixel intensity was 0.01 Relative Optical Density (ROD) units. To obtain counts of Fos-positive cells, a pixel intensity threshold of 0.03 RODs was set to allow for detection of cells that were three times the intensity of the background. During analysis, an image of Fos-positive cells and its corresponding MOR image were opened in two separate channels. A rectangular template was placed in the target region (DL and DM caudate-putamen, 1.34 mm^2^; nucleus accumbens core, 0.27 mm^2^; and nucleus accumbens shell, 0.20 mm^2^) of the MOR-stained image, and the software scanned the corresponding area in the Fos-stained image to obtain overall cell counts. To determine Fos density in specific compartments of the dorsal striatum, MOR-positive regions (“patches”) were distinguished from MOR negative regions (“matrix”) by using the Autoscan feature in MCID. This tool automatically detects an edge between a target and background when the target is clearly distinct from the surrounding regions. Since the signal intensity in patches is much greater than in the matrix, MCID was able to generate an outline of the patch with the remaining areas of the image labeled as the matrix. Cells in the corresponding Fos image were then counted in an area based on the outline of the patch. To calculate the density of Fos-positive cells in the matrix, the number of cells in the patch regions was subtracted from the total number of cells, and patch area was subtracted from the total area. Fos counts were expressed as the number of Fos-positive cells per mm^2^. Single pixel targets were automatically removed and other abnormal targets were deleted manually. An observer blind to the experimental conditions conducted all quantification.

### Statistics

For behavioral analysis, a mixed model ANOVA was performed to examine interactions between pretreatment, challenge, and time. For quantification of Fos expression, a mixed model ANOVA was performed to examine interactions between treatment, challenge, and rostral-caudal level or compartment. When no significant three-way interactions were observed, data were collapsed across the entire time course or rostral-caudal level. Two-way ANOVAs were then used to examine the effects of treatment and challenge. In cases where the treatment x challenge interaction reached significance, t-tests were used for pairwise comparisons. A Fishers exact test was used to analyze the effect of pretreatment on specific locomotor velocity ranges.
